# Diabetic Gait Is Not Just Slow Gait: Gait Compensations in Diabetic Neuropathy

**DOI:** 10.1155/2019/4512501

**Published:** 2019-11-11

**Authors:** Adrienne D. Henderson, A. Wayne Johnson, Sarah T. Ridge, Jonathan S. Egbert, Kevin P. Curtis, Levi J. Berry, Dustin A. Bruening

**Affiliations:** ^1^Exercise Sciences Department, Brigham Young University, Provo, UT, USA; ^2^Canyon Foot and Ankle, Spanish Fork, UT, USA

## Abstract

**Background:**

Neuropathic complications from diabetes mellitus affect multiple nerve types and may manifest in gait. However, gait compensations are still poorly understood, as narrow analyses and lack of speed controls have contributed to conflicting or equivocal results.

**Purpose:**

To evaluate gait mechanics and energetics in diabetic peripheral polyneuropathy.

**Methods:**

Instrumented gait analysis was performed on 14 participants with diabetic peripheral polyneuropathy and 14 matched controls, walking at 1.0 m/s. A full-body model with a multisegment foot was used to calculate inverse dynamics and analyze sagittal plane metrics and time series waveforms across stance phase.

**Results:**

Alterations included increased hip and knee flexion in early stance followed by a prolonged hip extension moment in midstance. Late stance ankle dorsiflexion and power absorption were increased, and final push-off was delayed and truncated.

**Conclusion:**

A neuropathic diabetic gait shares important similarities to a mild crouch gait with weakness/dysfunction in the foot and ankle. This study highlights two main compensation mechanisms that have been overlooked in previous literature. First, increased triceps surae stretch in terminal stance may be used to increase proprioception and/or energy storage, while a prolonged hip extension moment in midstance compensates for a limited push-off. These result in an overall workload shift from distal to proximal joints. Clinical assessment, monitoring, and treatment of neuropathy may benefit by focusing on these specific functional alterations.

## 1. Introduction

Neuropathic complications from diabetes mellitus can result in numerous functional deficits, particularly in the distal lower extremities. Diabetic peripheral polyneuropathy (DPN) afflicts up to 50% of people with diabetes and accounts for roughly 25% of the enormous total cost of diabetic care [1]. While DPN diagnosis and progression have traditionally been assessed using sensory tests [2, 3], DPN has been shown to affect all three nerve types (autonomic, sensory, and motor) [4, 5]. Damage to motor nerves, for example, can result in muscle atrophy and fatty infiltration, which in turn affects dynamic gait function [4]. With more than just sensation loss affecting DPN gait, many associated compensations are still not well understood. A better understanding of how these changes affect gait, energetics, and loading may help clinicians better treat the devastating effects of DPN.

Although DPN gait has received substantial attention in the literature, two major limitations are apparent. First, the vast majority of these studies have not evaluated the influence of walking speed. Early studies primarily evaluated spatiotemporal metrics at self-selected speeds and were useful in characterizing the slower, more cautious gait used by DPN patients [6] but did not focus on identifying specific joint contributions to this gait pattern. Only three studies that we are aware of controlled for speed in some form, with differing methodology. Bacarin et al. controlled cadence [7], Yavuzer et al. used a fast (1.4 m/s) speed [8], which can be challenging for DPN subjects, and DiLiberto et al. used a slow (0.9 m/s) speed [9, 10]. Gait speed can greatly affect joint mechanics [11]; for example, findings of decreased joint ranges of motion [8, 9, 12] in DPN during self-selected walking speeds are consistent with what would be expected simply by walking more slowly [11, 13]. Without speed controls, it is difficult to separate the influences of DPN on joint mechanics from those due to speed alone.

The second limitation is that most joint mechanics studies have analyzed only a narrow scope of variables. This has led to mixed or inconclusive results across studies, as illustrated in several reviews of DPN gait [6, 14–17]. Many studies, for example, measured only joint angles [18] or only joint moments [19]. Studies combining kinematics and kinetics have typically isolated only a single joint or region; for instance, DiLiberto et al. [9, 10] performed a thorough analysis of foot and ankle mechanics but did not include proximal joints. A focus on metrics rather than whole time series has also masked potential findings. For example, studies evaluating joint range of motion (RoM) across the gait cycle have primarily shown a decreased total ankle RoM in DPN [8, 9, 12]. However, time series graphs in Raspovic [20] and DiLiberto et al. [9] suggest potential increases in peak ankle dorsiflexion as well as time delays in subsequent plantarflexion. Hip joint kinetic results in particular have presented an intriguing conundrum. Both Mueller et al. [18] and Sacco et al. [21] have suggested that ankle weakness in DPN is overcome through increased internal hip flexor moments in late stance, theoretically to pull the body forward. However, Savelberg et al. [19] showed an increased hip extensor moment in midstance with subsequently decreased hip flexion moments. While both findings reflect potential hip compensations, they are diametrically opposite each other and are unlikely to exist together. The latter theory is more in line with previous research showing greater hip extensor contributions to support and forward progression in healthy gait [22].

A more comprehensive analysis of DPN gait mechanics may help consolidate findings across studies and identify gait compensations. Therefore, the purpose of the present study was to evaluate gait mechanics in DPN compared to healthy controls, accounting for speed and employing a comprehensive kinematic and kinetic analysis. We specifically hypothesized that DPN-afflicted individuals would manifest ankle weakness through reduced ankle and midfoot power generation, a delayed transition from dorsiflexion to plantarflexion, and compensate for this weakness by increasing hip extensor moments in midstance. A better understanding of DPN gait alterations may provide insight into the effects of DPN on motor function and assist clinicians in providing improved assessment and treatment.

## 2. Methods

### 2.1. Participants

Twenty-eight participants, fourteen with DPN and fourteen healthy matched controls (CON), participated in this case-control study (Table 1). DPN participants were screened for and excluded if they had a history of ulcers, amputation, and any neurological condition besides DPN or could not walk unassisted. Exclusion criteria for the control group (CON) included a history of diabetes, any type of peripheral neuropathy, arthritis, or any lower extremity injury in the past 6 months. All subjects were volunteers and signed informed consent forms approved by the local ethics board. The presence of DPN was confirmed using the Michigan Neuropathy Screening Instrument (MNSI) [23]. DPN participants were queried on whether one foot was more affected, and if identified, this side was targeted in the analysis. For equally affected feet and for all CON participants, a side was arbitrarily chosen by the researchers.

### 2.2. Protocol

A total of fifty-six reflective markers were affixed to each subject with double-sided tape according to a custom full-body model. Briefly, this consisted of upper extremity markers on the acromioclavicular joints, sternum, and 7th cervical spinous process to capture torso motion, with additional markers on the head, elbow, and wrist in order to record the center of mass (not presented in this study). A marker cluster was placed on the posterior pelvis with anterior superior iliac spine and posterior superior iliac spine landmarks identified using a digitizing pointer. Additional clusters were used to track the thighs and shanks with individual markers on the medial and lateral aspects of the knee and ankle. An additional 11 markers were placed on the selected foot, according to a 3-segment foot model modified slightly from Bruening et al. [24]. The contralateral foot employed a simple, single-segment foot model with four markers (heel, metatarsal heads, and dorsum).

Barefoot walking tests were performed on a walkway with two force plates (AMTI, Inc., Watertown MA, USA) embedded flush with the floor. A thin carpet was secured over the walking surface to protect participants' feet. Participants were first instructed to walk down the walkway at a natural, comfortable speed. Three trials were collected and used to determine each subjects' self-selected walking speed. Next, the subjects walked down the walkway at a controlled speed of 1.0 m/s. This speed was chosen as a midrange for subjects with DPN [6]. A motor-driven pulley system was used to help subjects maintain the desired speed, similar to that of Thompson et al. [25]. A waist-high string with small colored flags ran between two pulleys—participants simply matched the speed of the flags ahead of them as they walked. They were allowed to practice walking with the device at the controlled speed until they were consistently matching it. Each participant's starting position was adjusted during this practice period to ensure a full contact of the evaluated foot on one force plate, allowing them to walk as naturally as possible without targeting foot placement.

### 2.3. Data Analysis

A biomechanical model including the pelvis, thigh, shank, foot, head, torso, and upper and lower arm segments was created in Visual 3D software (C-Motion Inc., Germantown, MD, USA) according to common conventions while the 3-segment foot was made based off the model used by Bruening et al. [24]. The model included anatomically aligned rearfoot, mid-/forefoot, and phalange segments, separated by midtarsal and metatarsophalangeal (MTP) joints. In addition to anatomically aligned segments, additional kinematic-only, laboratory aligned versions of the pelvis and foot segment reference frames were created. The anatomical alignments were used to measure standing posture, while the laboratory alignments were used to measure dynamic motion.

Marker trajectories and force data were low-pass filtered at 6 Hz and 50 Hz, respectively. Joint angles were calculated based on a typical Euler/Cardan angle rotation sequence (1-sagittal, 2-frontal, and 3-transverse). Joint moment vectors and scalar power quantities were calculated using inverse dynamics. Moments were expressed as internal moments, resolved in the proximal segment reference frame. For the foot, midtarsal kinetics were calculated only when the center of pressure passed anterior to the midtarsal joint [26].

Metrics and time series were analyzed. Metrics consisted of demographics, spatiotemporal metrics (speed, cadence, and % stance) for both self-selected and controlled speed trials, and postural metrics extracted from the static pose. Postural metrics included pelvic tilt, anterior trunk lean, and midtarsal angle (a surrogate for arch height). These were included to determine whether DPN participants had increased forward leaning posture or decreased arch height, both of which could influence and help explain gait mechanic compensations. All metrics were compared between groups using independent *t*-tests (*α* = 0.05). Time series angles, moments, and powers were time-normalized to 100% of stance and averaged across the three trials for each subject. Aggregate group means and standard error bands were then plotted for visualization and descriptive analysis. Joint kinetics were normalized to body mass for comparisons. To focus the time series analysis to a manageable scope, in this study, we present only stance phase kinematics and kinetics and only sagittal plane angles and moments. In addition, only the controlled speed trials are presented. Finally, the total amount of positive and negative work (integral of power) performed at each lower extremity joint was calculated.

## 3. Results

The DPN and CON groups were well-matched demographically (Table 1) for age and height. Mean body mass was 10 kg higher in the DPN group, but this was not statistically significant (joint kinetics were also normalized to body mass). MNSI scores for DPN ranged from 2 through 11, with a mean score significantly higher than CON. All CON participants scored lower than the neuropathy cutoff of two. No differences were found in any of the standing postural metrics (Table 2).

At self-selected pace, DPN participants walked significantly slower than CON (Table 3). In order to match the controlled speed, DPN increased speed by 7% while CON decreased by 11%. No differences were found in the controlled speeds or time spent in stance between the groups (Table 3).

**Figure 1:**
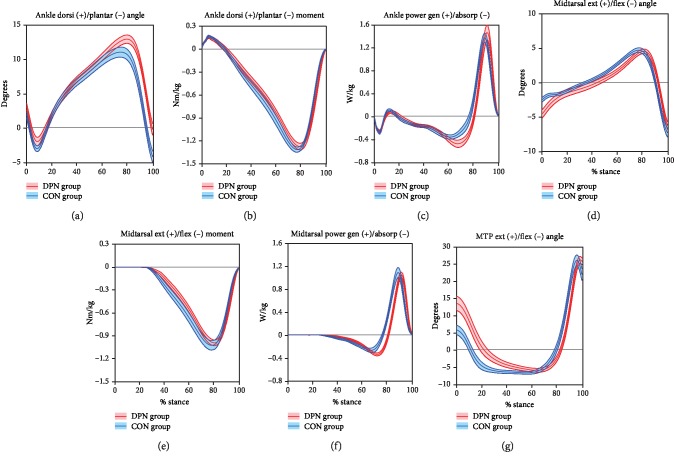
Ankle and foot mechanics. Mean ± SE bands for DPN and CON groups are displayed across time-normalized stance phase.

Ankle and foot joint mechanics (Figure 1) showed a few distinctions between groups, primarily in late stance timing. DPN exhibited increased dorsiflexion in late stance, followed by a delayed transition to plantarflexion. This was accompanied by slightly increased power absorption in the ankle and midtarsal joints. In all three of these measures, similar mild delays in the transition to power generation were also seen, as were slight delays in ankle and midtarsal moment production, midtarsal flexion, and MTP extension. Early stance mechanics were similar between groups, with the exception of slightly decreased plantarflexion and midtarsal flexion, and increased MTP extension in DPN.

**Figure 2:**
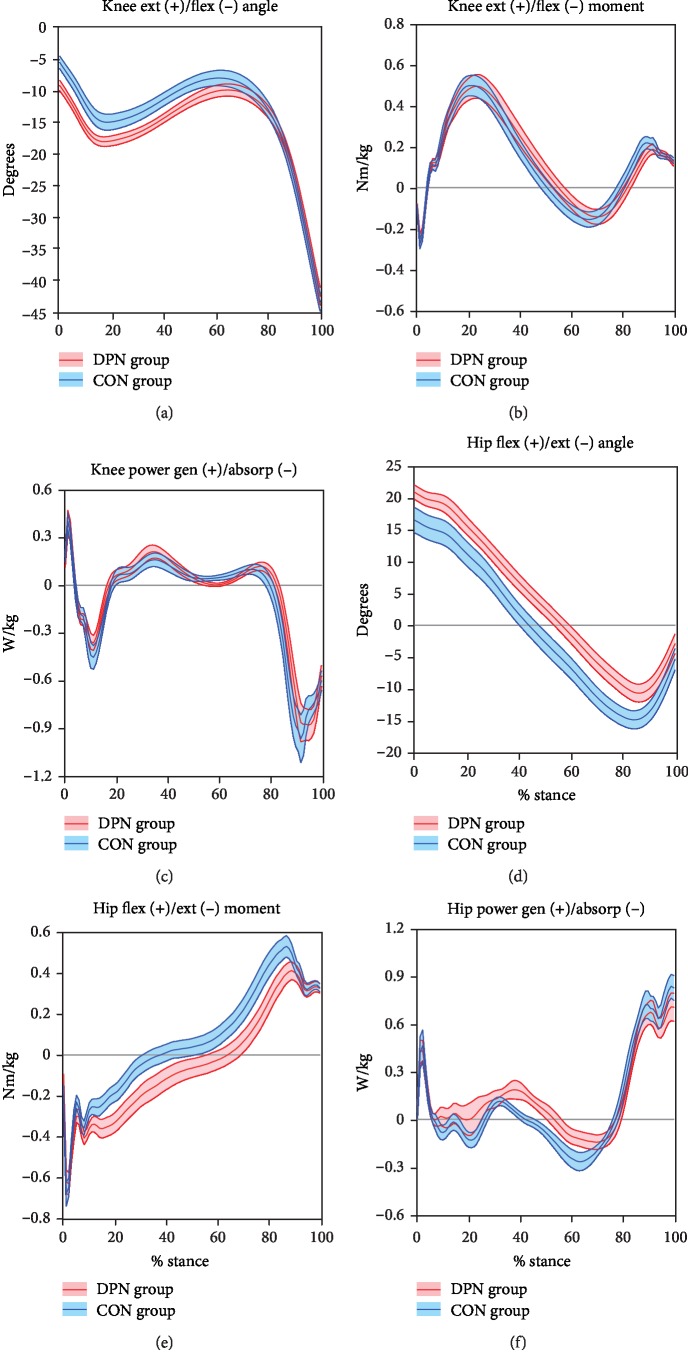
Knee and hip mechanics. Mean ± SE bands for DPN and CON groups are displayed across time-normalized stance phase.

Group distinctions in knee and hip mechanics were primarily found in early stance knee angles and midstance hip angles, moments, and power (Figure 2). At the knee, DPN exhibited increased knee flexion throughout early stance, yet knee kinetics were similar between groups. Hip flexion was also increased in DPN throughout most of stance. DPN exhibited a substantially prolonged hip extension moment in midstance, with an accompanying delay in the transition from hip extension to hip flexion moment. This also resulted in a slight decrease in hip flexion moment peak. Hip power showed concomitantly prolonged power generation in midstance and subsequently slightly reduced power absorption.

**Figure 3:**
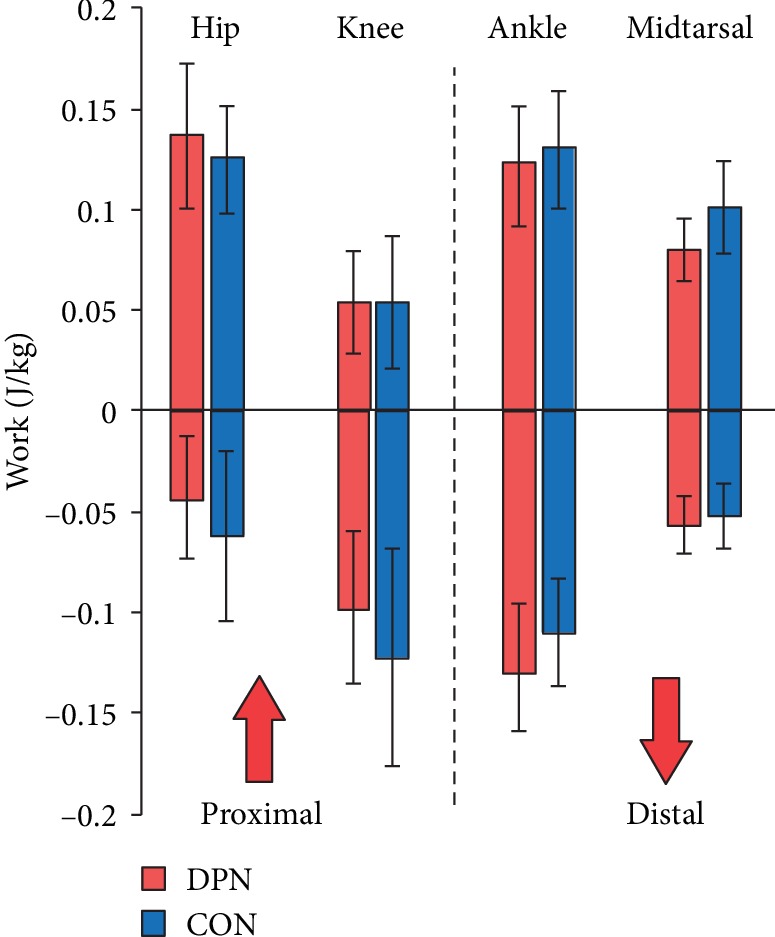
Joint and distal foot work. Mean ± standard deviations for DPN and CON groups.

While there was substantial variability in total positive and negative joint work of the hip, knee, ankle, and midtarsal joints, there was a general shift in the DPN group towards positive work in the proximal joints and towards negative work in the distal joints (Figure 3).

## 4. Discussion

The purpose of this study was to investigate gait mechanics alterations and compensations in individuals afflicted with diabetic peripheral polyneuropathy. Our metric results show close group matches in demographics as well as spatiotemporal and postural measures, allowing us to focus our synthesis on DPN-related influences to joint mechanics. Insights are discussed below, separated by early, mid-, and late stance phases.

### 4.1. Early Stance (Initial Contact through Loading Response)

Beginning at initial contact, the lower limb was in a mildly flexed posture (Figures 2(a) and 2(d)). This is in line with previous studies showing a more cautious style of gait in DPN [18] with increased lower limb flexion. This gait pattern contains similarities to a mild crouch gait seen in cerebral palsy and other pathologies that, although differing in etiology, result in distal weakness [27]. Distally, the increase in MTP extension during early stance in DPN (Figure 1(g)) was unexpected since no prior studies have included hallux kinematics. This finding could be related to weakness [28] or motor dysfunction [29] of the ankle dorsiflexors. The tibialis anterior acts concentrically during swing for foot clearance and eccentrically during loading response to control the rate of plantarflexion. Weakness in this muscle may be compensated for by excessive recruitment of synergists, such as the toe extensors. However, MTP extension was also increased in midstance when the tibialis anterior is normally quiet. DPN may contribute to an inability to relax the great toe extensors. Additional analysis including electromyography (EMG) may help elucidate this mechanism. The increased MTP extension may also have contributed to the measured increase in midtarsal flexion during early stance through premature activation of the windlass mechanism [30].

### 4.2. Midstance (Midstance through Early Terminal Stance)

Throughout midstance, the DPN hip appears to compensate for distal dysfunction by increased and prolonged hip extensor moments (Figure 2(e)). In healthy gait, the hip extensor muscles are active in early stance to provide body support, but quiet in midstance as momentum-driven passive dynamics take over [22]. When distal weakness is present, proximal muscles may compensate for insufficient momentum. In this case, hip extensor activity is likely increased and prolonged to aid in forward progression of the center of mass, similar to compensations in cerebral palsy [31] and immature children [32].

Once the center of mass passes anterior to the ankle, the plantar flexor muscles eccentrically control anterior tibial progression [33]. In DPN, the increase in dorsiflexion, dorsiflexion angular velocity (Figure 1(a)), and ankle power absorption (Figure 1(c)) suggests a minor collapse at the ankle joint instead of a controlled roll forward [33, 34]. This likely indicates greater reliance on passive restraints and an accompanying increase in Achilles tendon strain. It is possible that this strain benefits DPN individuals by engaging alternate proprioceptive mechanisms that compensate for compromised plantar surface afferent input [35]. Midtarsal joint mechanics show similarities to the ankle through early terminal stance, including increased power absorption (Figure 2(f)). However, there was not an increase in midtarsal dorsiflexion, which may be due to increased midfoot tissue stiffness [30].

### 4.3. Late Stance (Late Terminal Stance and Pre-Swing)

Late stance alterations can be characterized by a delayed and truncated final push-off. In healthy gait, eccentric plantarflexor control is followed by a short burst of power generation, typically initiated during terminal stance just prior to opposite foot weight acceptance. This positive work theoretically contributes to both whole body and swing leg acceleration [36]; however, since plantarflexor EMG muscle activity is typically waning at this time, much of this power is theorized to arise from elastic energy storage and return [37, 38]. In DPN, this power generation is delayed and truncated, yet peak power generation was similar between groups. It is possible that DPN subjects utilize a delayed but forceful muscular response; however, previous EMG studies on DPN do not show atypical gastrocnemius activation timing [12, 39]. More likely, the increased prior strain from dorsiflexion could result in greater musculotendon energy storage and subsequent release. If so, it would be in spite of decreasing tendon elasticity [40] and likely heavily involve muscle fiber strain [41]. At the midtarsal joint, positive power was more delayed and truncated than at the ankle. This could be due to weakness of the smaller extrinsic and intrinsic foot muscles [42, 43], which may be active later in stance than the gastrocnemius [36], and/or decreased engagement of the windlass mechanism. The latter is apparent in the delayed onset of MTP extension and reduced peak ankle plantarflexion, suggesting potentially reduced passive power transfer from the MTP joint to the midtarsal joint [44].

### 4.4. Synthesis and Applications

Overall, our results show a shift in DPN energy use and muscle contributions from distal to proximal joints, as well as increased reliance on passive structures in the foot and ankle. All of these compensations are consistent with peripheral muscle weakness and dysfunction, with the hip extensors compensating for a decreased ability to control the second rocker and generate propulsive power at the ankle. This energy shift can be seen in the total positive and negative work done at each joint (Figure 3). While not dramatic, taken across all joints, there is a subtle shift in net work done from the distal ankle and midtarsal joints to the proximal knee and hip.

The increased use of proximal muscles, primarily the hip extensors, appears to be an effective compensation for distal weakness. Our hip moment graphs are similar to those of Savelberg et al. [19], but markedly different from Mueller et al. [18], Sacco et al. [21], and Santos et al. [45], all of whom showed decreased hip extension moments in midstance and increased hip flexion moments in late stance. It is not clear why the discrepancy between studies exists; however, it is difficult to see how increased hip flexion moments in late stance could effectively aid in advancing the body center of mass [22]. Our results are more in line with compensations found in other gait patterns that exhibit distal muscle weakness or dysfunction, such as immature children [32] or cerebral palsy [31]. In particular, the increased knee and hip flexion in early stance and increased hip extension moments in midstance are similar to a mild crouch gait [27, 31]. Several studies on this gait pattern show that the hamstrings are activated to a greater extent and later into mid- and late stance as a compensation to maintain body support and forward propulsion [31, 46, 47]. Understanding proximal compensations may be helpful clinically, for example, in fall prevention [48]. While rehabilitation including strengthening, motor control, or functional training of the whole lower extremity is likely ideal, in the presence of compromised distal motor control, a focus on proximal muscles may be beneficial.

While our foot and ankle results are mostly consistent with previous literature, they reveal new insights. Prior studies have focused only on the reduced overall range of motion in DPN. Our results suggest that this reduction arises primarily from truncated pre-swing plantarflexion; yet, it is the prior increase in dorsiflexion during terminal stance that likely has a greater effect on gait energetics. This alteration can be seen in waveforms from previous studies [9, 20] but has received little attention. DiLiberto et al. [9, 10] identified similar foot and ankle power changes, suggesting that the greater negative to positive power ratio at the ankle and midfoot could lead to foot pathologies. These kinematic and kinetic alterations may have other clinical implications, for example, contributing, through high strains [41], to connective tissue and even further muscle degeneration [40]. In addition, a lack of control over the second rocker likely compromises weight transfer and may contribute to instability and fall risk. Targeting this mechanism may inform fall prevention strategies. It is also possible that increased ankle and midtarsal power absorption is related to the increased forefoot plantar pressure often seen in DPN [15, 49]. A better understanding of the relationship between gait and pressure may ultimately help inform ulcer rehabilitation and prevention interventions (e.g., combining DPN footwear recommendations with gait alterations, such as the collapse at the ankle and increased great toe extension).

Our results also highlight a few future study recommendations. Monitoring the extent of the distal to proximal muscle strategy in longitudinal or cross-sectional samples of varying DPN severity may help determine the functional progression of DPN. Investigating the relationship between tissue stiffness [50] and elastic energy storage/return [38] could help clarify energy utilization in DPN. EMG of smaller foot muscles may also be of benefit in clarifying active foot muscle contributions.

### 4.5. Limitations

There were several limitations in this study. First, the use of a controlled speed may have induced additional gait changes in both DPN and CON groups. However, a number of factors suggest that these effects were minimal. The 1.0 m/s speed was chosen as a midrange speed and required only mild speed changes to both groups. Spatiotemporal metrics also showed similar gait timing between groups. In addition, our waveforms match several amalgamated previous results that were performed at a variety of speeds. Overall, we felt that this speed minimized speed effects in both groups, allowing us to better isolate the effects of DPN. For manageability, we analyzed only sagittal plane kinematics. Some results from other planes are available in isolated studies, but deviations are minor compared to the sagittal plane. Another limitation is the lack of a separate group with diabetes but without neuropathy [21]. We did not include this group because differences between DPN and diabetic samples are subtle in comparison to those between DPN and controls. However, we cannot conclude with certainty that the results are due solely to neuropathy and not to diabetes itself. Future studies should investigate diabetes with and without neuropathy to further elucidate potential differences between these two groups. We also used only a limited survey to verify neuropathy in the participants. However, the purpose of this survey was simply to confirm the presence of neuropathy for study inclusion, rather than as a measurement variable. Our overall study goal was to investigate gait compensations used by high functioning DPN participants (i.e., initial stages of neuropathy).

## 5. Conclusions

By controlling for walking speed, we were able to isolate gait compensations in DPN. Whole-body modeling and time-series analysis elucidated several gait alterations that have either been missed or received insufficient attention in previous literature. Across the gait cycle, alterations can be summarized into three main important insights. First, DPN gait shares similarities with mild crouch gait, which may be an important cross-pathology recognition. Second, an uncontrolled second rocker is a marker of dysfunction but may also indicate compensation by increasing proprioception and/or passive energy storage and return. Third, distal muscle weakness results in a shift in joint workload from distal to proximal. Specifically, the limited ankle push-off is compensated for by a prolonged hip extension moment. We hope that identifying these alterations may help connect the effects of sensory and motor nerve degeneration and help clinicians and researchers better understand and treat the devastating effects of DPN.

## Figures and Tables

**Table 1:** Group demographics.

	DPN (*n* = 14)	CON (*n* = 14)	*p* value
Height (cm)	177.28 ± 7.78	177.99 ± 6.71	0.799
Weight (kg)	103.39 ± 10.08	92.74 ± 15.19	0.103
Age (yrs)	61.43 ± 12.44	61.64 ± 9.79	0.960
MNSI	6.36 ± 2.79	0.29 ± 0.49	<0.001^∗^

∗ indicates a significant difference between groups.

**Table 2:** Standing posture metrics.

	DPN	CON	*p* value
Pelvic tilt (deg)	11.0 ± 6.3	12.1 ± 6.0	0.640
Forward trunk lean (deg)	2.8 ± 4.5	1.0 ± 3.4	0.257
Midfoot angle (deg)	18.5 ± 4.6	18.3 ± 4.5	0.917

∗ indicates a significant difference between groups.

**Table 3:** Spatiotemporal metrics.

	DPN	CON	*p* value
Self-selected speed (m/s)	0.94 ± 0.15	1.15 ± 0.16	<0.001^∗^
Controlled speed (m/s)	1.00 ± 0.08	1.02 ± 0.06	0.481
% stance	62.5 ± 1.8	61.7 ± 1.1	0.148

∗ indicates a significant difference between groups.

## Data Availability

The motion and force data used to support the findings of this study are available from the corresponding author upon reasonable request.
